# Clinical and Therapeutic Profile of Dravet Syndrome in Patients Followed at a Brazilian Pediatric Referral Center: Real-World Data from a Tertiary Service

**DOI:** 10.3390/jcm15208034

**Published:** 2026-10-16

**Authors:** Lisandra Coneglian de Farias Rigoldi, Daniel Almeida do Valle, Mayla Cristine de Souza, Maria Augusta Kormann, Solena A. Fidalski, Mara Lucia S. F. Santos, Mara L. Cordeiro

**Affiliations:** 1 Faculdades Pequeno Príncipe, Curitiba 80230-020, PR, Brazildaniel.valle@hpp.org.br (D.A.d.V.); 2 Hospital Pequeno Príncipe, Curitiba 80250-060, PR, Brazil; pediatriamayla@gmail.com (M.C.d.S.);; 3 Instituto de Pesquisa Pelé Pequeno Príncipe, Curitiba 80250-060, PR, Brazil; 4 Department of Psychiatry and Biobehavioral Sciences, David Geffen School of Medicine, University of California Los Angeles, Los Angeles, CA 90025, USA

**Keywords:** epilepsy, developmental and epileptic encephalopathy, pharmaco-resistant epilepsy, *SCN1A*, Dravet syndrome, real-world evidence

## Abstract

**Background:** Dravet syndrome (DS) is a severe developmental and epileptic encephalopathy, and data from low- and middle-income countries remain scarce. We characterized the clinical, genetic, and therapeutic profiles of patients with DS at a Brazilian tertiary referral center. **Methods:** This retrospective cross-sectional study included 27 patients with clinically and genetically confirmed DS (pathogenic/likely pathogenic *SCN1A* variants). Outcomes were ≥6-month and ≥12-month seizure freedom and seizure control in the previous month, described by treatment exposure without inferential testing. **Results:** The median age at seizure onset was 4 months and at diagnosis 42 months; the median individual onset-to-diagnosis interval was 29 months (range, 4–283; *n* = 14). All patients received polytherapy; 51.9% used cannabidiol, 40.7% vagus nerve stimulation, and 7.4% fenfluramine. Only 2/20 patients (10.0%) achieved ≥6-month and 1/20 (5.0%) ≥ 12-month seizure freedom. All six patients seizure-free in the previous month were receiving valproate, a descriptive finding likely reflecting confounding by indication. Recorded comorbidities, mainly autism spectrum disorder (29.6%), were likely underestimated in the absence of systematic neurodevelopmental assessment. **Conclusions:** This Brazilian DS cohort showed marked diagnostic delay, high seizure burden, and limited access to DS-specific therapies. These findings support earlier genetic testing and broader access to recommended treatments and generate hypotheses for larger prospective studies.

## 1. Introduction

Dravet syndrome (DS) is a rare and severe developmental and epileptic encephalopathy characterized by early-onset, prolonged seizures, frequently triggered by fever in previously healthy infants [1]. During the first years of life, additional seizure types typically emerge, including myoclonic seizures and focal impaired consciousness seizures. Over time, most patients develop drug-resistant epilepsy, associated with developmental delays or regression, behavioral disturbances, and motor impairments, including gait abnormalities [2]. DS is recognized as a multisystem disorder with neurological and behavioral manifestations beyond epilepsy, significantly impacting quality of life [3,4].

In >80% of cases, DS is caused by pathogenic *SCN1A* gene variants. *SCN1A* encodes the α-subunit of the voltage-gated sodium channel NaV1.1, a key regulator of neuronal excitability [5]. However, *SCN1A* variants are associated with a broad phenotypic spectrum ranging from febrile seizures to severe epileptic encephalopathies. Less frequently, variants in other genes, such as *SCN2A*, *GABRA1*, and *STXBP1*, have been reported in DS-like phenotypes [6,7,8].

Emerging evidence suggests that DS may progress into adulthood, with reports of functional decline, Parkinsonian features, and cognitive deterioration in later decades of life [9].

Therapeutic management remains challenging owing to pharmaco-resistance and the need for tailored treatment strategies. Diagnosis is often delayed as the initial manifestations may overlap with other pediatric epilepsy types, hindering timely therapy initiation. Recently, novel precision medicine-based approaches, including gene-targeted therapies and antisense oligonucleotides, have shown promise in modifying disease progression [10].

Given DS’s clinical heterogeneity and complexity, studies characterizing its clinical and genetic profiles in various populations are essential. Data from low- and middle-income countries remain scarce, particularly regarding the clinical presentation and therapeutic outcomes. In Brazil, published data have focused mainly on the molecular interpretation of *SCN1A* variants [11] or on caregiver-reported non-seizure symptoms [12], whereas clinician-documented data on diagnostic pathways, treatment patterns, and seizure outcomes remain limited. Such data are relevant because diagnostic delay and access to DS-specific therapies depend on health-system characteristics that differ from those of the high-income settings in which most DS cohorts have been described [13]. Therefore, this study aimed to characterize the clinical, genetic, and therapeutic profiles of pediatric and young-adult patients with DS followed at a Brazilian tertiary referral center, to quantify diagnostic delay and describe access-related treatment patterns in this setting, and to describe, in a hypothesis-generating manner, the relationship between treatment strategies and seizure outcomes.

## 2. Materials and Methods

### 2.1. Study Design

This observational, descriptive, retrospective study analyzed the epidemiological, genetic, and phenotypic profiles of pediatric and young-adult patients diagnosed with DS at a large tertiary referral pediatric hospital in Curitiba, Paraná, Brazil.

### 2.2. Participants

Patients aged 0–32 years, with a confirmed clinical and genetic DS diagnosis, were included. Individuals aged >18 years at the time of data collection had been followed up continuously since childhood, allowing characterization of the transition from pediatric to adult care.

Clinical diagnosis was established according to consensus criteria from an expert panel, including (i) seizure onset within the first year of life; (ii) prolonged seizures, often triggered by fever; (iii) evolution to multiple seizure types; (iv) developmental slowing or regression after seizure onset; and (v) exclusion of alternative structural or metabolic etiologies.

Molecular confirmation was based on the identification of pathogenic or likely pathogenic *SCN1A* gene variants, classified according to the American College of Medical Genetics and Genomics and Association for Molecular Pathology (ACMG/AMP) guidelines [14].

Patients with alternative genetic epileptic syndromes or inconclusive diagnoses were excluded.

For descriptive purposes, participants were stratified into three groups according to therapeutic approach:

- Group 1: patients exclusively treated with antiseizure medications (ASM);
- Group 2: patients who underwent vagus nerve stimulation (VNS);
- Group 3: patients receiving ketogenic diet (KD).

Patients in Groups 2 and 3 also received ASM per current clinical practice. Cannabidiol (CBD) was analyzed as an exposure variable across groups. Treatment allocation reflected clinical decisions made during routine care and was not randomized.

Clinical outcomes related to seizure control were defined using two complementary measures derived from medical records. The primary outcome was ≥6-month seizure freedom, defined as a seizure-free period of at least 180 consecutive days. This threshold was adopted as a pragmatic operational definition for this retrospective study: given the high seizure burden typical of DS (in this cohort, the median seizure-free interval was 12.5 days), a 6-month seizure-free period represents a clinically meaningful change that could be ascertained from medical records. It is, however, less stringent than the International League Against Epilepsy (ILAE) definition of seizure freedom and is not directly comparable with studies applying that criterion. The ILAE criterion for seizure freedom used in the definition of drug-resistant epilepsy requires a seizure-free period of at least 12 months or three times the longest pre-treatment inter-seizure interval, whichever is longer [15]; however, this criterion could not be reliably applied owing to incomplete and variable pre-treatment seizure data. To improve comparability, the proportion of patients who were seizure-free for ≥12 months is also reported. The secondary descriptive outcome was seizure control in the previous month, defined as the absence of seizures in the month preceding the evaluation. This outcome was retained as a secondary descriptive measure of short-term clinical stability; because it is a single-time-point assessment, it is strongly influenced by the natural fluctuation of seizures in DS and was not used to infer treatment effects. Outcome analyses were restricted to patients with complete seizure frequency data.

### 2.3. Study Procedure

Data collection involved a retrospective review of patient medical records at a specialized service. The epidemiological variables included sex, current age, age at diagnosis (in months), family history of epilepsy or other neurological diseases, presence of associated comorbidities, and clinical course during follow-up.

Phenotypic characterization encompassed clinical aspects such as epileptic seizure type and frequency, response to instituted therapies, and psychomotor development, according to information available on record. Seizure types were recorded as documented in the charts and are reported descriptively. For the 21 patients with detailed variant annotation, whose records were individually re-reviewed, the documented seizure types and the circumstances of seizure onset (including onset following vaccination) were extracted. Fever sensitivity and history of status epilepticus were not systematically documented in the records and are therefore not reported. Motor (head control, sitting, and walking) and language (first words/phrases) milestones were recorded to characterize neurodevelopment.

### 2.4. Genetic Evaluation

All patients underwent genetic investigation using either multigene panel testing (including *SCN1A* among 229 genes overall) or whole-exome sequencing, depending on availability at diagnosis. Genetic analyses were performed at Mendelics Laboratory (Brazil). DNA samples obtained from peripheral blood or buccal swabs were processed using next-generation sequencing on an Illumina platform. Sequencing reads (FASTQ format) were aligned to the human reference genome (GRCh38). Variant calling was performed after removing polymerase chain reaction (PCR) duplicates and low-quality reads using standard bioinformatics pipelines, including Burrows–Wheeler Aligner and SAMtools.

Variants were filtered based on quality metrics (consensus quality > 30, single-nucleotide polymorphism (SNP) quality > 30, and root-mean-square mapping quality > 30). Regions with low sequencing coverage and candidate variants were confirmed using Sanger sequencing.

Variants were described according to the Human Genome Variation Society (HGVS) nomenclature and classified per ACMG/AMP guidelines. Novel variants were evaluated using allelic frequencies, evolutionary conservation, segregation analysis, and in silico prediction tools. Databases, such as the Human Gene Mutation Database (HGMD) and ClinVar, were used for cross-referencing. Variants of uncertain significance were reclassified, when possible, based on clinical correlations and family studies.

### 2.5. Statistical Analyses

Data were organized in Microsoft Excel^®^ and analyzed using IBM SPSS Statistics^®^, version 27.0. Categorical variables are summarized as absolute and relative frequencies. Continuous variables with non-normal distribution are reported as median and range. Seizure outcomes were described according to exposure to each treatment (valproate, CBD, VNS, and KD) and age at diagnosis was described by variant class. No inferential comparisons between treatment or variant subgroups are reported: only two patients achieved ≥6-month seizure freedom, subgroups were very small (e.g., *n* = 2 for KD), all patients received polytherapy, and treatment allocation was driven by clinical decisions. Under these conditions, *p*-values would be dominated by low statistical power, multiplicity, and confounding by indication (e.g., baseline seizure severity, disease duration, treatment duration, number of concomitant medications, or previous treatment failures), which could not be adjusted for. All comparisons are therefore descriptive and hypothesis-generating and cannot be interpreted as evidence of causal or comparative treatment effects.

## 3. Results

### 3.1. Patient Demographic and Baseline Characteristics

Relevant patient characteristics are reported in Table 1. Twenty-seven patients with a clinical and genetic DS diagnosis, confirmed by *SCN1A* gene variant identification, were included.

### 3.2. Phenotypic Profile

In the analysis of epileptic seizure types, classified according to the ILAE 2025 terminology [16], generalized tonic–clonic seizures predominated and were present in most patients. Epileptic spasms, focal impaired consciousness seizures, and focal preserved consciousness seizures were also observed. Among the 19 of 21 re-reviewed patients with documented seizure types, generalized tonic–clonic seizures were recorded in 15 (78.9%) and focal to bilateral tonic–clonic seizures in 1 (5.3%); focal seizures, either motor or with impaired consciousness, were recorded in 6 (31.6%), and myoclonic seizures, atonic seizures, and behavioral arrest in 1 patient each (categories are not mutually exclusive). Seizure onset was recorded as following vaccination in 4 of the 16 patients (25.0%) with documented circumstances of onset. Descriptive semiological manifestations included head deviation, clonic limb movements, and unilateral eye blinking episodes. Seizure frequency varied widely, from multiple daily seizures (up to 450/month) to weekly, monthly, and twice-yearly occurrences.

Analysis of clinical outcomes stratified by the two operational definitions showed a marked contrast between short-term stability and longer seizure-free periods. According to the last-month control definition, 6 of the 21 patients with available data (28.6%) had no seizures in the month immediately preceding the assessment. Only 2 of the 20 patients with quantitative seizure-free time data (10.0%) achieved ≥6-month seizure freedom, and only 1 patient (5.0%) was seizure-free for ≥12 months, the threshold closest to the ILAE criterion. The median seizure-free time was 12.5 days (range, 0–365 days), consistent with the syndrome’s high epileptic burden and refractoriness. The approximately three-fold discrepancy between the two definitions illustrates the clinical instability typical of DS, in which periods of apparent stability are frequently followed by seizure recurrence, and indicates that single-time-point measures overestimate durable seizure control.

Recorded comorbidities included neurodevelopmental and systemic clinical conditions. The distribution of recorded comorbidities is presented in Table 1. These figures reflect comorbidities documented in the medical records rather than systematically ascertained prevalence. The proportion of patients without described comorbidities (55.6%) likely does not reflect an actual absence of associated conditions but rather ascertainment bias related to limitations in access to formal neuropsychological assessment in the public health service. This gap most likely resulted in underdiagnosis of neurodevelopmental disorders, including intellectual disability, in this population, as discussed below. Regarding developmental milestones, systematic characterization of head control, sitting, walking and language acquisition was not uniformly available in all charts, which precluded specific statistical analyses of these milestones—an aspect detailed in the Section 4.7.

### 3.3. Genetic Profile

Molecular analysis revealed considerable heterogeneity regarding *SCN1A* gene variant types, including missense (*n* = 8), nonsense (*n* = 4), frameshift (*n* = 4), splice-site (*n* = 3), and in-frame deletions (*n* = 2). In the remaining six patients, the variant was not classified into one of these molecular types: one patient carried a 2q24.3 microdeletion (described below), and in five, the genetic report confirmed a pathogenic alteration in *SCN1A* compatible with the clinical diagnosis, but the detailed molecular description (predicted effect on the protein) was not recorded. These five patients were not included in the variant-class analyses or in Figure 1. From a functional standpoint, 11 and 8 patients harbored predicted loss-of-function (nonsense, frameshift, and splice-site) and missense variants, respectively; the two in-frame deletions were not assigned to either category, and the whole-gene microdeletion is described separately. In one patient, a 689-kb microdeletion was identified in the 2q24.3 region, encompassing the contiguous *SCN1A* and *SCN2A* genes and consistent with a contiguous gene deletion syndrome within the spectrum of sodium channelopathies. Figure 1 shows the distribution of variants along the NaV1.1 protein, stratified by molecular type and protein domain (DI–DIV).

#### Age at Diagnosis by Variant Class (Descriptive)

Descriptively, among patients with available age at diagnosis, the median age at diagnosis was 24 months in the carrier of an in-frame deletion (*n* = 1), 27 months in carriers of frameshift variants (*n* = 3), 39.5 months in carriers of nonsense variants (*n* = 4), 79.5 months in carriers of missense variants (*n* = 8), and 84 months in carriers of splice-site variants (*n* = 3). Given the very small subgroups, no formal comparison was performed, and these data do not support a genotype–phenotype association; age at diagnosis is also influenced by non-genetic factors, such as healthcare access, clinical awareness, and availability of genetic testing.

### 3.4. Pharmacological Treatment Profile

All patients were on polytherapy with concomitant use of two or more antiseizure medications; the exact number of concomitant medications and of previous treatment lines per patient was not systematically available. Heterogeneity was observed regarding associated therapies: some patients were maintained exclusively on pharmacological polytherapy, whereas others received combined adjunctive therapies such as CBD, VNS, or KD. Overall, 14 patients (51.9%) received CBD, 11 (40.7%) underwent VNS, and 2 (7.4%) received KD. VNS duration, stimulation parameters, and adverse events were not systematically documented in the records and are therefore not reported.

The most frequently used antiseizure medications were clobazam and sodium valproate, commonly used in combination. Other medications included topiramate (*n* = 4; 14.8%), stiripentol (*n* = 4; 14.8%), levetiracetam (*n* = 10; 37.0%), and fenfluramine (*n* = 2; 7.4%).

### 3.5. Treatment Exposure and Seizure Outcomes (Descriptive Analyses)

Because only two patients achieved ≥6-month seizure freedom, this outcome was not analyzed according to treatment exposure. Regarding seizure control in the previous month, all six seizure-free patients were receiving valproate (6/13 valproate users [46.2%] vs. 0/8 non-users; Figure 2). Given the non-randomized allocation, universal polytherapy, and absence of adjustment for disease severity, treatment duration, or concomitant medications, this observation should not be interpreted as evidence of a therapeutic effect of valproate.

Seizure control in the previous month was observed in 5/14 CBD users (35.7%) and 1/7 non-users (14.3%), in 2/11 patients with VNS (18.2%) and 4/10 without VNS (40.0%), and in 1/2 patients receiving KD (Figure 2). The median monthly seizure count was 10 in the VNS group and 53 in the non-VNS group. Given the small subgroups and the non-random allocation of these therapies, which are typically added in more refractory patients, these descriptive differences do not allow inferences about benefit or its absence.

Seizure outcomes were not compared across variant types, loss-of-function status, or affected protein domains, because these subgroups were too small for meaningful comparison.

Figure 2 shows seizure control in the previous month according to exposure to each treatment. Because several combinations of variant class and treatment included fewer than three patients or none, outcomes were not further stratified by variant class.

## 4. Discussion

The present study characterizes the genotypic, phenotypic, and therapeutic profiles of patients with Dravet syndrome followed at a Brazilian pediatric tertiary referral center. The findings highlight the substantial clinical and molecular heterogeneity of DS in this population, as well as the persistent burden of pharmaco-resistant epilepsy and the frequent need for polytherapy and adjunctive therapeutic strategies. Pathogenic *SCN1A* variants showed considerable diversity in type and distribution, while treatment patterns reflected the complexity of long-term clinical management. Many of these features are well established in DS; the contribution of this study lies in documenting how they manifest in a Brazilian tertiary-care setting. Specifically, it (i) quantifies a diagnostic delay substantially longer than that recently reported in European centers [13]; (ii) shows that single-time-point seizure control markedly overestimates longer seizure-free periods, with only 10% of patients achieving ≥6-month seizure freedom; (iii) documents access-related treatment patterns, including frequent use of VNS and CBD alongside limited use of fenfluramine and stiripentol; and (iv) includes young adults followed continuously since childhood. These real-world data add to the limited clinician-documented evidence on DS from middle-income countries.

### 4.1. Diagnosis and Care Pathway

The first international consensus on DS, published in 2022 [17], with the aim of promoting early diagnosis and improving clinical outcomes, recommends that infants aged between 2 and 15 months presenting with a first prolonged hemiclonic seizure or convulsive status epilepticus associated with fever or occurring after vaccination, in the absence of another identifiable cause, should undergo genetic investigation for DS. In the present cohort, seizure onset was recorded as following vaccination in 4 of the 16 patients with documented circumstances of onset, illustrating a presentation that, according to these recommendations, should prompt early genetic testing.

Despite these recommendations, the median age at diagnosis in this study was 42 months (mean, 71.5 months), and the median individual interval between seizure onset and diagnosis was 29 months (range, 4–283 months; *n* = 14). By comparison, the multicenter European DS’coverED study reported a median age at diagnosis of 15 months and a median interval of 11 months between seizure onset and diagnosis [13]. In an earlier Argentine series, the diagnosis was usually established only after 2–3 years, once the electroclinical picture was complete [18]. Diagnostic delay is often related to the initial variability in epileptic manifestations and their resemblance to simple febrile seizures, which frequently hinder early suspicion. In the DS’coverED study, delays were additionally attributed to late referral to expert centers, low awareness in acute-care settings, and variable access to and turnaround time of genetic testing [13]. In the Brazilian context, likely contributors include limited recognition of DS warning signs in primary and emergency care, fragmented referral pathways to pediatric neurology, and unequal access to genetic testing across the public and private health systems [12]. Because the retrospective data did not allow reconstruction of the referral pathway for each patient, these factors could not be quantified in the present cohort.

Expanded access to multigene panels and exome sequencing is expected to shorten the interval between symptom onset and diagnosis; however, the present study was not designed to assess whether diagnostic delay decreased over time with the broader availability of genetic testing, and this question should be addressed in larger cohorts stratified by period of diagnosis. Early genetic confirmation allows the avoidance of sodium channel blockers and other potentially aggravating medications and the timely introduction of recommended therapies [17]. In the Brazilian setting, practical measures may include (i) structured training of primary-care and emergency pediatricians on the consensus-based red flags for DS; (ii) a fast-track genetic testing pathway for infants aged 2–15 months with prolonged, hemiclonic, or fever- or vaccine-associated seizures, in line with the international consensus [17]; and (iii) formal referral pathways to reference centers within the National Policy for Comprehensive Care of People with Rare Diseases [19].

### 4.2. Genotype–Phenotype Correlations

The genotype–phenotype correlation in DS is recognized as complex and involves the type and location of *SCN1A* gene variants, their functional impact, and the possible influence of genetic modifiers. Previous studies [20,21] indicate that variants resulting in complete loss of function of the NaV1.1 sodium channel (truncating variants and deletions) tend to be associated with more severe clinical phenotypes. Variants with partial loss of function, such as missense variants, are usually associated with less severe presentations, although exceptions are frequent [20,21].

In the present study, variants were distributed across the four homologous domains (DI–DIV) of NaV1.1. Importantly, as demonstrated by Nissenkorn et al. [22], patients harboring the same missense variant may display heterogeneous phenotypes, suggesting the involvement of additional genetic modifiers and the influence of individual genomic contexts on the clinical expression of the disease. Two patients in this study carried the c.4934G>A (p.Arg1645Gln) variant, one of whom had a controlled clinical picture after VNS, whereas the other maintained focal seizures and an associated autism spectrum disorder (ASD). This phenotypic variability in two unrelated patients carrying the same variant is consistent with the hypothesis that the variant alone does not capture the effect of genetic or environmental modifiers.

In the present cohort, differences in age at diagnosis across variant classes were descriptive only, and no formal comparison was performed given the very small subgroups. These data therefore do not support a genotype–phenotype association. Moreover, age at diagnosis is strongly influenced by non-genetic factors, including healthcare access, clinical awareness, and the availability of genetic testing at the time of presentation, which may outweigh any effect of variant class in a small retrospective series. Larger multicenter cohorts with systematic phenotyping would be required to examine whether variant class influences age at diagnosis in the Brazilian setting.

Seizure outcomes were not compared across variant classes in the present cohort because of the very small subgroups. In a larger cohort, Zuberi et al. [23] reported only a modest genotype–phenotype correlation, limited to sub-analyses by protein domain. In a South Indian cohort of 49 children, seizure freedom was more frequent among carriers of missense than truncating variants [24], suggesting that genotype-related differences may emerge in larger samples. The small sample size of the present series, the absence of detailed annotation in five patients, and the lack of systematic phenotypic data precluded stratification by degree of functional impairment or protein domain and the comparison of other phenotypic dimensions, such as seizure types, seizure severity, and neurodevelopmental comorbidities, across variant classes.

### 4.3. Treatment Exposure and Seizure Outcomes: Valproate, CBD, VNS, and KD

All treatment–outcome observations in this study are descriptive. The finding that all patients who were seizure-free in the previous month were receiving valproate is compatible with the position of valproate as a first-line medication in international recommendations [17,25], but it cannot be interpreted as evidence of effectiveness. Treatment was not randomized, all patients received polytherapy, and no adjustment was possible for baseline seizure severity, disease duration, treatment duration, number of concomitant medications, or previous treatment failures. Confounding by indication is likely: patients with more refractory disease are more likely to have valproate replaced or supplemented by add-on therapies, such as CBD, VNS, or KD, whereas those with milder disease are more likely to remain on conventional regimens. In addition, this observation is based on a single-time-point outcome and on only six patients.

In this cohort, 5 of 14 CBD users (35.7%) and 1 of 7 non-users (14.3%) were seizure-free in the previous month; these small numbers do not allow any inference about CBD effectiveness, which has been established in pivotal randomized clinical trials of CBD in DS, such as the GWPCARE1 and GWPCARE2 studies, which demonstrated a significant reduction in convulsive seizure frequency compared with placebo [26,27]. The absence of a clear pattern in our data can be explained by (i) the observational and cross-sectional nature of the present study, with heterogeneity in time of use and dose; (ii) the use of CBD in patients more refractory to conventional pharmacological treatment, introducing indication bias; and (iii) sample limitations.

The median monthly seizure count was lower in the VNS group than in the non-VNS group (10 vs. 53), whereas seizure control in the previous month was less frequent among VNS users (2/11 vs. 4/10). Given the small subgroups, the likely allocation of VNS to more refractory patients, and the absence of data on VNS duration and stimulation parameters, these descriptive observations do not allow inferences about benefit. Evidence from randomized trials in drug-resistant focal epilepsy supports the efficacy of VNS [28]; seizure freedom with VNS is uncommon, although response rates tend to increase over time [29]; and a meta-analysis of 68 patients with DS reported seizure reduction with adjunctive VNS in a proportion of patients [30]. Conversely, Mithani and Ibrahim [31] have cautioned against the progressive normalization of neuromodulation practices supported by limited evidence, including pediatric applications, and advocated for systematic registries capturing both efficacy and safety outcomes. The relatively frequent use of VNS in this cohort (40.7%) may also reflect the limited availability of DS-specific medications in Brazil. Two patients in the VNS group had a marked reduction in seizure frequency, shifting from daily seizures to sporadic episodes; these individual observations are descriptive and do not allow conclusions about the effectiveness of VNS in DS.

KD was used in only two patients, both of whom had a reduction in seizure frequency recorded during follow-up; this sample is too small for any inference.

Fenfluramine use was observed in only two patients (7.4%) in this study, despite the robust evidence of its efficacy in DS demonstrated in pivotal trials [32,33], reflecting specific barriers in the Brazilian context, even in a tertiary referral center. Fenfluramine was approved by the U.S. Food and Drug Administration (FDA) in 2020 and, more recently, by the Brazilian Health Regulatory Agency (ANVISA) for the treatment of DS-associated seizures in patients aged ≥2 years [34]. Nevertheless, factors limiting its widespread incorporation into national clinical practice persist, including high costs, lack of incorporation into the Unified Health System (Sistema Único de Saúde, SUS), no routine coverage by private insurance, and access frequently depending on legal action (judicialization). A similar situation applies to stiripentol, used by four patients (14.8%) in this cohort: it was registered by ANVISA only in April 2026 and, at that time, was not yet commercially available in Brazil [35]; until then, access depended on importation, frequently through legal action. These barriers may delay or prevent the use of the therapies with the strongest evidence for DS [17,32,33] and may partly explain the high rates of CBD and VNS use observed in this cohort. Because the dates and routes of access to these medications were not systematically recorded, the time to access could not be analyzed.

### 4.4. Comparison with Cohorts from Other Low- and Middle-Income Settings

Clinical series from low- and middle-income settings share several features with the present cohort. In Argentina, Caraballo and Fejerman described 53 patients in whom seizures were difficult to control; all had cognitive delay, and the diagnosis was usually established only after 2–3 years of age; 13 of 20 patients treated with KD showed some degree of seizure control [18]. In a South Indian cohort of 49 children, polytherapy was the rule, 24 of the *SCN1A* variants identified were previously unreported, and seizure freedom was observed only among carriers of missense variants [24]. In Brazil, molecular data from 21 probands with DS have been used to refine the interpretation of *SCN1A* missense variants [11], and a national caregiver survey of 140 individuals with DS reported intellectual disability in 92.1% and autism spectrum disorder in 57.3%, with most families depending, at least partially, on the private health system for access to care [12]. Together with the present findings, these studies indicate that prolonged diagnostic delay, reliance on polytherapy, limited access to DS-specific medications, and incomplete assessment of neurodevelopmental comorbidities are recurrent features in resource-constrained settings. Because the present data derive from a single tertiary center, their generalizability to other Brazilian regions and health-system contexts is limited.

### 4.5. Comorbidities and Neurodevelopment

Since the initial description by Charlotte Dravet, epileptic seizures have been recognized to frequently follow a pharmaco-resistant course. Concurrently, cognitive alterations and developmental difficulties tend to emerge during childhood [36]. Regarding DS, specifically, these include intellectual disability, attentional difficulties, motor alterations, and sleep disorders [37].

In this study, 44.4% of the patients had at least one recorded comorbidity, with ASD being the most frequent (29.6%). These proportions reflect documentation in medical records rather than systematically ascertained prevalence and should not be interpreted as prevalence estimates. The proportion of patients without recorded comorbidities (55.6%) was very likely overestimated because the study did not apply standardized neuropsychological assessments, and not all patient records contained systematic descriptions of motor and language milestones. When information on developmental milestones was available, delays in head control, unsupported sitting, walking, and first words were observed in a substantial proportion of patients, including four cases with explicit descriptions of psychomotor developmental delay or gait impairment (wheelchair use). The recorded frequency of neurodevelopmental comorbidities was markedly lower than that reported in a Brazilian national caregiver survey, in which intellectual disability and autism spectrum disorder were reported in 92.1% and 57.3% of individuals with DS, respectively [12]. Because intellectual disability, ASD, and motor impairment are core features of DS, this discrepancy most likely reflects insufficient assessment and documentation rather than a true difference in prevalence.

Natural history studies, such as the prospective multicenter ENVISION study [4], have shown that behavioral and socioemotional alterations emerge early in children with pathogenic *SCN1A* gene variants, frequently before age 3 years. The most commonly described manifestations include inattention, social withdrawal, and features compatible with ASD. These data reinforce the hypothesis that such manifestations are a part of the neurodevelopmental phenotype associated with the disease, rather than merely secondary consequences of epileptic seizures [4]. The findings of this study are consistent with this profile, although they are limited by the heterogeneity of the records.

Recent evidence also suggests the possibility of progressive changes across the lifespan, including a neurodegenerative component described in adult patients [9,38], as well as heterogeneous trajectories of seizure frequency and speech progression documented in large European registry cohorts [39], reinforcing the importance of longitudinal studies that follow patients from early childhood into adulthood.

### 4.6. Therapeutic Approach

Historically, phenobarbital was used for the prophylaxis of febrile seizures in infants with early seizure onset, although this practice is now uncommon. With increasing knowledge regarding the molecular basis of DS, particularly its association with *SCN1A* gene variants, certain medications have been found to exacerbate neuronal excitability and worsen the seizure course in these patients. Consequently, a progressive shift in therapeutic strategies has been observed, with greater use of medications recommended in international guidelines, such as sodium valproate, clobazam, topiramate, and CBD.

This shift is expected to contribute to more targeted and safer approaches to DS management, with potential improvements in seizure control and reductions in adverse events. Considering DS’s pharmaco-resistant nature, future studies should focus on evaluating combinations of currently available therapies to optimize treatment strategies and improve clinical outcomes [17].

### 4.7. Limitations and Future Perspectives

This study has several methodological limitations. First, the single-center, retrospective, and cross-sectional design limits generalizability, introduces potential selection bias toward more severe cases referred to a tertiary center, and prevents a longitudinal analysis of clinical manifestations, particularly the evolution of behavioral and socioemotional alterations throughout development. Second, treatment allocation was not randomized and was driven by clinical decisions; confounding by indication is therefore likely, and no adjustment was possible for baseline seizure severity, disease duration, treatment duration, number of concomitant medications, or previous treatment failures. Third, the small sample size, with very small subgroups (e.g., *n* = 2 for KD and only two patients achieving ≥6-month seizure freedom), precluded meaningful inferential testing; treatment- and genotype-related comparisons were therefore restricted to descriptive analyses, and numerical differences should not be interpreted either as treatment effects or as evidence of their absence. Fourth, the number of possible subgroup comparisons was large relative to the sample size, so that any statistically significant result would have been prone to chance findings; this was a further reason for not reporting inferential tests. Fifth, the primary outcome (≥6-month seizure freedom) is a pragmatic endpoint that is less stringent than the ILAE definition of seizure freedom, which limits comparability with other studies, and the secondary outcome (seizure control in the previous month) is a single-time-point measure sensitive to the natural fluctuation of seizures. Sixth, several clinical variables were not systematically documented in the records, including fever sensitivity, history of status epilepticus, the number of concomitant medications and previous treatment lines, VNS duration, stimulation parameters, and adverse events, and the referral pathway of each patient; these data could therefore not be reported or analyzed. Seventh, the absence of standardized neuropsychological assessments and the lack of systematic documentation of developmental milestones—including head control, unsupported sitting, walking, and language acquisition—across all patient records precluded a comprehensive characterization of the neurodevelopmental phenotype. This documentation gap likely resulted in ascertainment bias and underestimation of the true prevalence of associated comorbidities, including intellectual disability, behavioral difficulties, and motor delays, as not all patients had access to formal neuropsychological evaluation within the public health service. Eighth, the absence of uniform neuroimaging data restricted detailed analyses of structural correlates of clinical progression. Finally, in five patients (18.5%), a detailed variant description was not available, which, together with the small number of patients per variant class, limited genotype–phenotype analyses to descriptive comparisons.

Prospective natural history studies conducted in multicenter cohorts, such as the ENVISION study [4], have demonstrated the importance of systematic longitudinal follow-up to identify the early emergence and progression of neurobehavioral manifestations. Prospective multicenter Brazilian investigations may contribute to characterizing the clinical evolution of the disease more precisely in our setting, guiding earlier, personalized therapeutic interventions.

Despite these limitations, this study provides a relevant real-world descriptive basis for the clinical, genetic, and therapeutic aspects of DS in Brazil. Multicenter studies integrating clinical, genetic, and neuropsychological data will expand our understanding of the pathophysiological mechanisms associated with DS and contribute to the development of targeted therapeutic strategies.

## 5. Conclusions

Our findings highlight persistent delays in DS diagnosis despite early seizure onset, underscoring the need for improved clinical recognition and broader access to timely genetic testing. This Brazilian cohort showed substantial phenotypic and molecular heterogeneity and a high burden of drug-resistant seizures, with only 10% of patients achieving ≥6-month seizure freedom. Neurodevelopmental comorbidities were likely underestimated, reinforcing the importance of systematic assessment and multidisciplinary long-term care. Limited access to DS-specific therapies, including fenfluramine and stiripentol, further illustrates challenges in DS management in resource-constrained settings. Earlier diagnosis and more equitable access to evidence-based therapies are essential to improve care for individuals with DS.

## Figures and Tables

**Figure 1:**
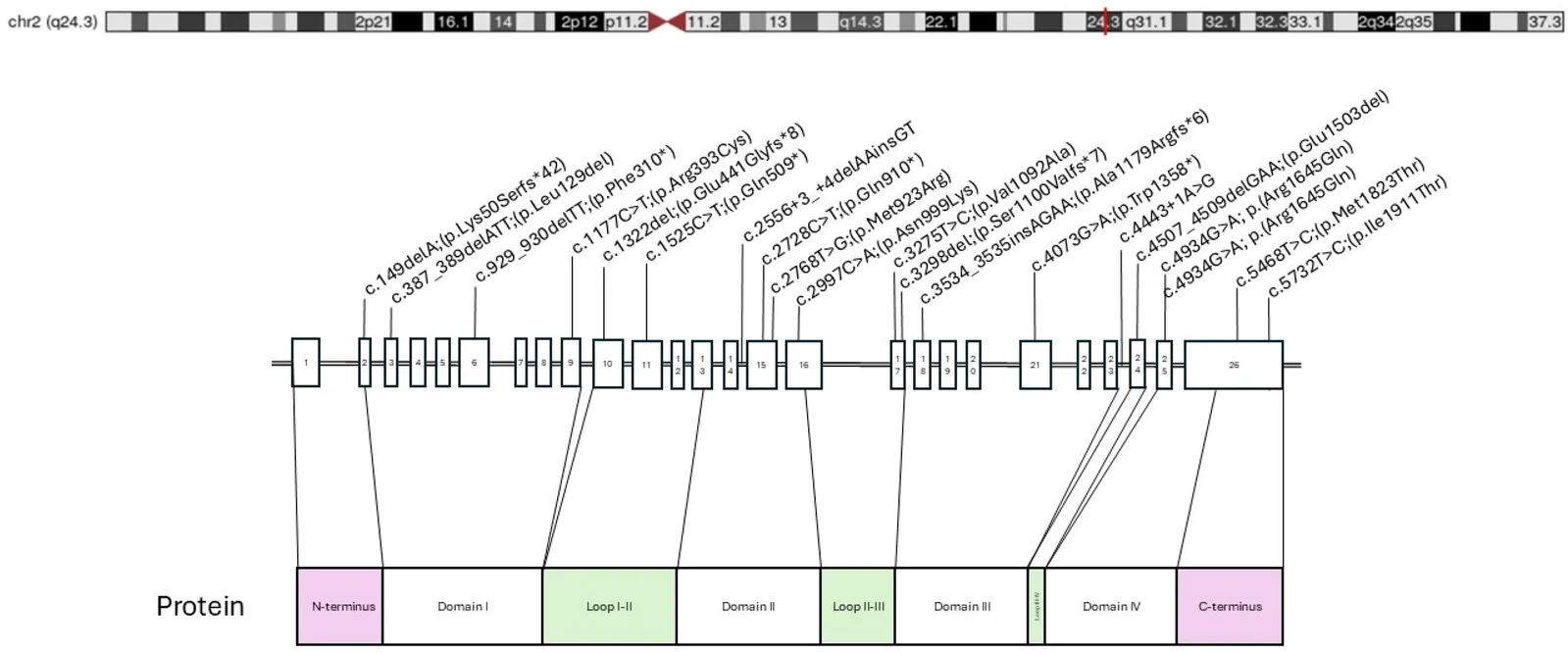
*SCN1A* pathogenic variant distribution along the NaV1.1 protein in patients with Dravet syndrome and detailed variant annotation, including the 2q24.3 microdeletion. The five patients without a detailed molecular description are not represented. Each variant is mapped to its corresponding position within the NaV1.1 protein, stratified by molecular type (missense, nonsense, frameshift, splice-site, and in-frame deletion) and homologous transmembrane domain (DI–DIV). Domain boundaries are based on the established topology of the NaV1.1 sodium channel α-subunit. The contiguous 2q24.3 microdeletion encompassing *SCN1A* and *SCN2A* is indicated separately. Abbreviations: DI–DIV, homologous domains I–IV of the NaV1.1 α-subunit; LOF, loss-of-function.

**Figure 2:**
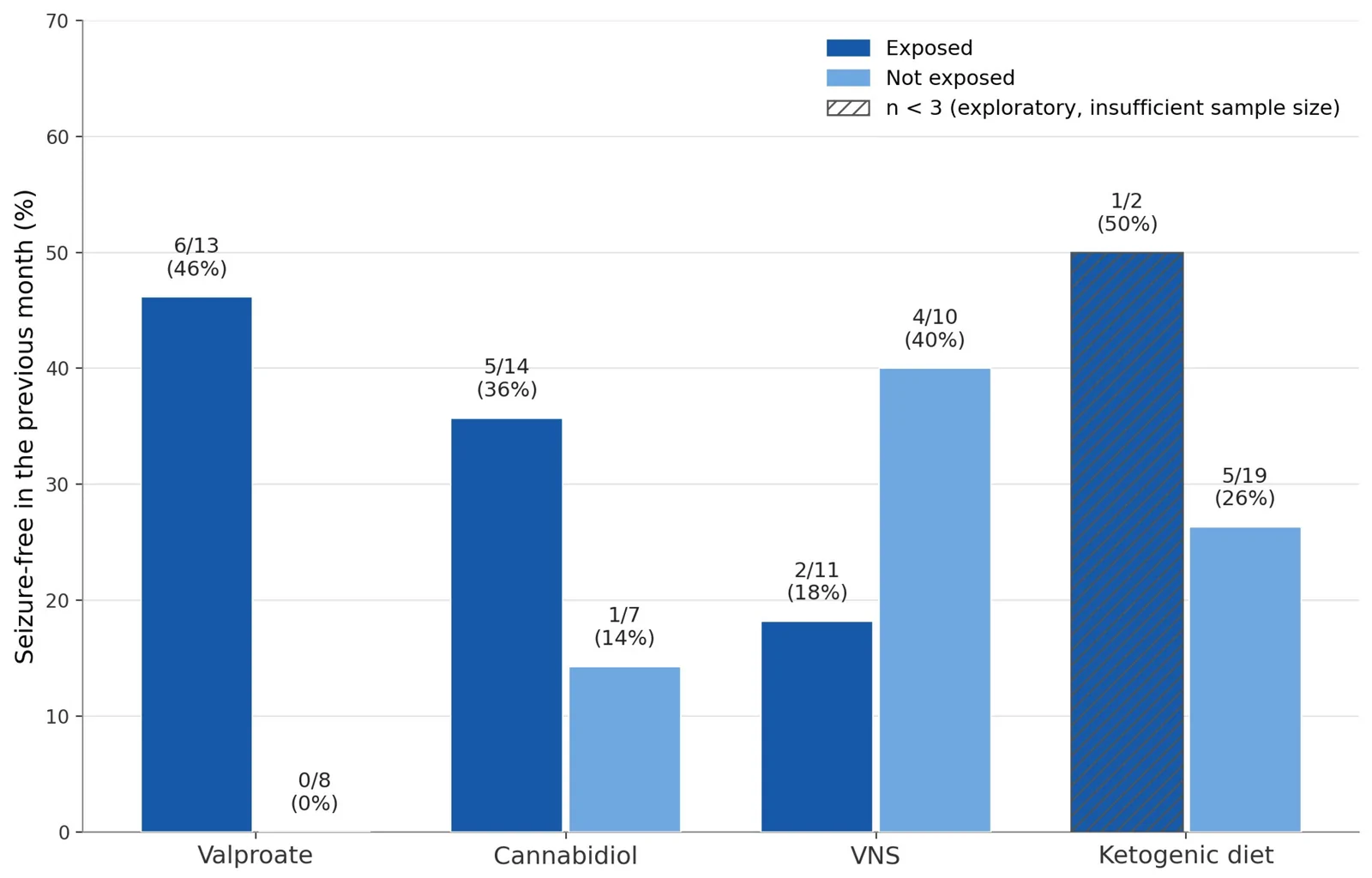
Proportion of patients without seizures in the previous month according to treatment exposure (*n* = 21 patients with available outcome data). Labels show the number of seizure-free patients/number of patients in each group and the corresponding percentage. Treatment categories are not mutually exclusive, because all patients received polytherapy; therefore, a patient may contribute to more than one treatment. The hatched bar (KD, *n* = 2) represents an exploratory result with insufficient sample size. Data are descriptive; no statistical comparisons were performed. Abbreviations: VPA, sodium valproate; CBD, cannabidiol; VNS, vagus nerve stimulation; KD, ketogenic diet.

**Table 1:** Demographic, genetic, therapeutic, and comorbidity profiles of patients with Dravet syndrome (n = 27).

Characteristic	*n* (%) or Median (Range)
**Demographics**	
Female sex	15 (55.6)
Age at seizure onset, months	4 (1–8)
Age at diagnosis, months–median (range)	42 (8–320)
Age at diagnosis, months–mean	71.5
Onset-to-diagnosis interval, months–median (range) ^a^	29 (4–283)
***SCN1A*** **variant type**	
Missense	8 (29.6)
Nonsense	4 (14.8)
Frameshift	4 (14.8)
Splice-site	3 (11.1)
In-frame deletion	2 (7.4)
2q24.3 microdeletion (*SCN1A*–*SCN2A*)	1 (3.7)
Variant type not specified in the genetic report	5 (18.5)
**Antiseizure medications**	
Patients on polytherapy	27 (100)
Clobazam and sodium valproate ^b^	Most frequent
Levetiracetam	10 (37.0)
Topiramate	4 (14.8)
Stiripentol	4 (14.8)
Fenfluramine	2 (7.4)
**Adjunctive/non-pharmacological therapies**	
Cannabidiol	14 (51.9)
Vagus nerve stimulation	11 (40.7)
Ketogenic diet	2 (7.4)
Comorbidities recorded in medical records ^c^	
Autism spectrum disorder (ASD)	8 (29.6)
IgA deficiency	1 (3.7)
Hypogammaglobulinemia	1 (3.7)
Hypothyroidism	1 (3.7)
Hyperammonemia	1 (3.7)
No comorbidities recorded	15 (55.6)

^a^ Calculated for each patient as age at diagnosis minus age at seizure onset; available for the 14 patients in whom both ages could be verified. ^b^ Clobazam and sodium valproate were the most frequently prescribed ASMs, commonly used in combination. ^c^ Comorbidities as documented in medical records; no standardized neurodevelopmental or neuropsychological assessment was applied, so these proportions are subject to ascertainment bias and do not represent prevalence estimates. Abbreviation: ASM, antiseizure medication.

## Data Availability

The de-identified dataset supporting the findings of this study is available from the corresponding author on reasonable request, subject to institutional data-sharing regulations. Genetic variant data have been deposited in ClinVar under accession numbers VCV001381930.11, VCV000495243.1, VCV003384846.4, VCV000068527.63, VCV001458263.9, VCV000068505.60, VCV000068558.25, VCV000206829.11, VCV001686148.6, VCV001075775.10, VCV000572287.46, SUB16376658, SUB16377761 and VCV003634033.2.

## Abbreviations

The following abbreviations are used in this manuscript:

**ACMG/AMP**	American College of Medical Genetics and Genomics/Association for Molecular Pathology
**ANVISA**	Brazilian Health Regulatory Agency
**ASD**	Autism spectrum disorder
**ASM**	Antiseizure medication
**CBD**	Cannabidiol
**DI–DIV**	Homologous domains I–IV of the NaV1.1 α-subunit
**DS**	Dravet syndrome
**FDA**	U.S. Food and Drug Administration
**HGMD**	Human Gene Mutation Database
**HGVS**	Human Genome Variation Society
**ILAE**	International League Against Epilepsy
**KD**	Ketogenic diet
**LOF**	Loss-of-function
**PCR**	Polymerase chain reaction
**SNP**	Single-nucleotide polymorphism
**SUS**	Sistema Único de Saúde (Brazilian Unified Health System)
**VNS**	Vagus nerve stimulation
**VPA**	Sodium valproate
